# Exploring a Moderate Fallow Scale of Cultivated Land in China from the Perspective of Food Security

**DOI:** 10.3390/ijerph16224329

**Published:** 2019-11-06

**Authors:** Dan Lu, Yahui Wang, Qingyuan Yang, Huiyan He, Kangchuan Su

**Affiliations:** 1 School of Geographical Sciences, State Cultivation Base of Eco-agriculture for Southwest Mountainous Land, Southwest University, Chongqing 400715, China; ludswu@email.swu.edu.cn (D.L.); wangyhui.15b@igsnrr.ac.cn (Y.W.); hhy1219@email.swu.edu.cn (H.H.); skc1986@email.swu.edu.cn (K.S.); 2 Research Base of Karst Eco-environments at Nanchuan in Chongqing, Ministry of Nature Resources, School of Geographical Sciences, Southwest University, 2 Tiansheng Rd, Chongqing 400715, China

**Keywords:** fallow scale, fallow system, food security, land-use change, population carrying capacity model, China

## Abstract

Food security remains a primary concern because of the large population and scarce land resources in China, and it is a core task to determine the appropriate proportion and scale of fallowing for fallow systems. The aim of this study was to systematically estimate the grain production potential (GPP) of existing and unexcavated cultivated land due to land use change from 1990 to 2017 and calculate the theoretical fallowing scale by using a population carrying capacity model. The reserved GPP from cultivated land to be excavated was 7470 × 10^4^ t in China, and the GPP stored by the change in grain yield per unit, multiple crop index (MCI) decline, and agricultural structure adjustment were 921 × 10^4^ t, 4321 × 10^4^ t, and 7760 × 10^4^ t, respectively, and the lost GPP caused by construction land expansion was 5287 × 10^4^ t. The population carrying capacity of cultivated land in China was estimated to be 1.469 to 1.515 billion in 2017 on the basis of the national average living standard. The proportion of the population that could be fed more was between 6.28% and 9.54% depending on the number of people included, which provided an opportunity to implement the fallowing system in China. Meanwhile the proportions of the theoretical fallow scale were 6.28% and 9.54%, and the fallow scale ranged from 850 × 10^4^ hm^2^ to 1296 × 10^4^ hm^2^ under the premise of fully tapping the potential of cultivated land. In addition, taking the decline in MCI as an example, the grain yield reduction was equivalent to the grain yield of 829 × 10^4^ hm^2^ of newly reclaimed cultivated land over the past 30 years, which saved 621.48 billion yuan. The costs and benefits when formulating relevant policies of land utilization should be considered, and exploiting the productive capacity of cultivated land that exists is better than reclaiming new cultivated land.

## 1. Introduction

The grain output of China has increased for 12 consecutive years since 2004, and the grain production capacity has greatly improved [1]. However, the ecological environment has paid a large price, such as in the thinning of the black soil layer [2,3], soil erosion [4], soil acidification [5,6], excessive heavy metals in soil [7], and increased non-point source pollution [8,9]. Meanwhile, construction land continues to expand, and the corresponding cultivated land area decreases yearly with the rapid development of urbanization [10,11,12]. The quality and quantity of cultivated land in China are declining to varying degrees, and great challenges exist for increasing the grain supply under the dual pressures of increasing pollution in agriculture and future construction land expansion [11,13].

To solve ecological environmental problems such as land degradation, the Chinese government issued a pilot programme for exploring and implementing cultivated land rotation and fallow systems in 2016 [14,15]. Subsequently, Central Committee No.1 documents have been proposed for three consecutive years to further expand the pilot scale of cultivated land rotation and fallow systems. China has implemented cultivated land rotation and fallow pilot programmes in groundwater funnel areas, heavy metal contaminated areas, and degraded ecological environmental areas [15,16], and since that time, the fallow scale has continuously expanded, from 41 × 10^4^ hm^2^ in the initial stage to 80 × 10^4^ hm^2^ in 2017. In 2019, the pilot rotation and fallow scales supported by the central government were further expanded to 200 × 10^4^ hm^2^, in which the fallow area was 34 × 10^4^ hm^2^. The fallow system has yet to be implemented throughout the country, although pilot programmes have been implemented in 15 provinces, such as Inner Mongolia, Liaoning, Jilin, and Heilongjiang. However, food security has always been a focus of national concern [17]. Considering the large population and scarce land resources in China, determining a reasonable fallow scale of cultivated land under the premise of ensuring food security is a key issue that needs to be solved immediately.

Regarding the abovementioned issue, some scholars have explored the fallow scale of cultivated land from the perspective of food security, but there are large differences in the understanding of fallow scale [18,19]. Some studies reported that the total fallow scale of cultivated land in China should be controlled within 5% to 8%, with a maximum proportion not exceeding 20% [18,19]. Wang et al. discussed the theoretical fallow scale in China on the basis of the prioritization of food security and suggested that the appropriate fallow scale was 400 × 10^4^ hm^2^ [17]. Moreover, Luo and Zou determined a base planting area of 1.08 × 10^8^ hm^2^; the authors believed that the proportion of fallow land should be controlled at 6–8% and that the fallow scale limit in China should be 2730 × 10^4^ hm^2^ [20]. Thus, the difference in the fallow scale estimated by the two abovementioned papers is 6.83 times.

However, the previously mentioned studies had some limitations, mainly concerning the fallow scale of cultivated land [17,18,21]. First, most of the studies focused on theoretical analyses and lacked accurate data for systematic calculations, making it impossible to make scientific judgements regarding the current fallow scale. Second, existing studies used the minimum per capita cultivated land area or the population carrying capacity of land as the basis to statistically estimate the fallow area and did not consider land use change. With the development of the social economy and urbanization, many labourers have moved from rural areas to cities in the past 30 years in China [22]. A series of changes have taken place in cultivated land, such as extensive land management [23,24], agricultural structure adjustment [25], and cropland abandonment in rural China [22,26]. Changes in cultivated land use patterns will inevitably have an impact on national food security, especially reductions in the multiple crop index (MCI) [24] and cropland abandonment in mountainous areas [22]. MCI is the ratio of total sown area and cropland area in a region, which represents the regional time intensity of planting crops. Therefore, we must fully consider land use change when determining a reasonable fallow scale, that is, the reserved and lost grain production potential (GPP) caused by cultivated land use change being estimated to understand the productive capacity of cultivated land in China.

On the basis of the abovementioned analysis, the purpose of this study was to systematically estimate the reserved and lost GPP from existing and unexcavated cultivated land in China from the aspects of grain yield change, MCI reduction, agricultural structure adjustment, and construction land expansion since the 1990s. Furthermore, a reasonable fallow scale of cultivated land was calculated by using a panel data model and a population carrying capacity model based on food security. This study is structured as follows. Section 2 describes land use change and GPP in China since the 1990s. Section 3 presents the materials and methods. Section 4 presents the results. Section 5 is the discussion, and Section 6 presents conclusions and implications. Our results were compared with other studies and documents required by the Chinese government, and our findings will enhance the understanding of land use change in China and serve as a scientific reference for the implementation and improvement of fallow systems in China.

## 2. Land Use Change and the Reserved and Lost GPP in China 

With continuous increases in non-agricultural wages, many rural labourers have migrated to cities. The national average annual reduction in the agricultural labour force was approximately 11.33 million from 2000 to 2015 [27]. Consequently, a series of changes have taken place in cultivated land use in rural China, such as a decline in the MCI [24], agricultural structure adjustment [25], and the occupation of cultivated land by construction land [10,11,12,28], and these changes have undoubtedly had a great impact on the GPP [29]. Land-use change can be divided into reversible and irreversible cultivated land use change.

### 2.1. Reversible Cultivated Land Use Change

Reversible cultivated land use change means that although the grain production capacity of cultivated land has decreased, the production capacity can be recovered. This type can be regarded as grain stored in the ground, which mainly includes grain yield per unit decrease caused by the extensive operation of cultivated land, a decline in MCI, and a reduction in grain yield due to agricultural structure adjustment. 

#### 2.1.1. Reserve of GPP Due to a Decline in Cultivated Land Yield per Unit

The labour intensity of cultivated land has decreased continuously since 1999, from 2.59 individuals per hm^2^ in 1999 to 2.12 individuals per hm^2^ in 2012, with an average annual decline of 1.53%. Intensive cultivated land use and the grain yield per unit decreased in this process [30]. Taking Guangdong Province as an example, the grain yield per unit fell from 5835 kg per hm^2^ in 1999 to 5496 kg per hm^2^ in 2012 according to data from the National Bureau of Statistics, and the reduction in grain yield was approximately 86.11 × 10^4^ t based on the estimated grain sown area in 2012. Importantly, grain output reduction caused by extensive cultivated land operation was not due to the loss of the grain production capacity from cultivated land, but the output could be increased by increasing intensive operations if necessary.

#### 2.1.2. Reserve of GPP Due to a Reduction in MCI

Since the 1990s, the MCI of staple crops has declined. Taking rice as an example, its MCI decreased from 148.3% in 1990 to 129.3% in 2015, a decrease of 14.7%. In southern China, large-scale double cropping rice was converted to single cropping rice; the lost sown area of grain was 253.16 × 10^4^ hm^2^, and the grain output was reduced by 2.6% [24]. Similarly, the grain output reduction caused by the decline in MCI does not mean that the cultivated land lost part of its grain production capacity, but the output can still be increased by increasing the MCI if necessary.

#### 2.1.3. Reserve of GPP Due to Agricultural Structure Adjustment

**Figure 1:**
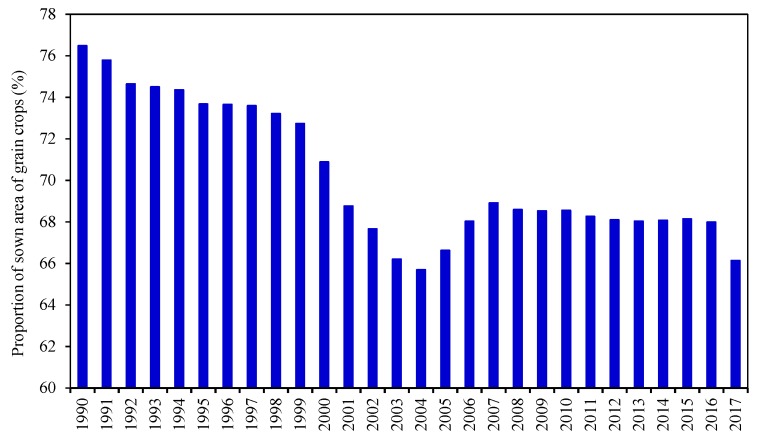
Proportion of the sown area of grain crops from 1990 to 2017.

The food consumption structure of residents has changed greatly with the development of the social economy, especially increased demands for meat, vegetables, and fruits, which has adjusted the agricultural structure [31]. According to the National Bureau of Statistics of China, the proportion of the sown area of grain crops declined between 1990 and 2017 (Figure 1), from 76.48% in 1990 to 66.13% in 2017, a decline of 9%.

**Figure 2:**
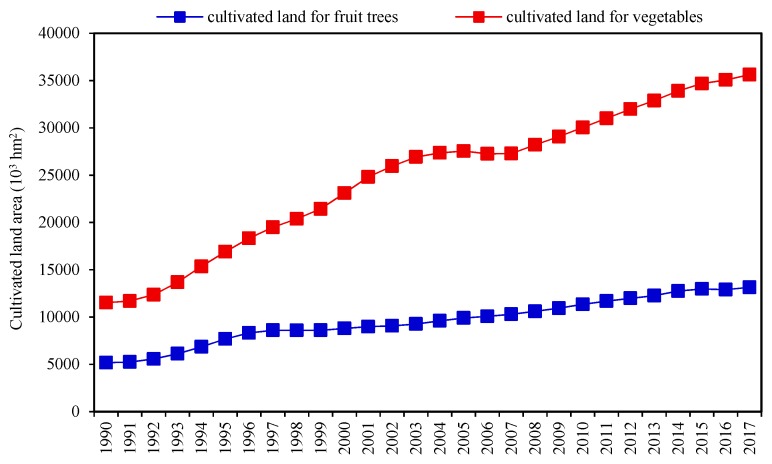
Cultivated land area for fruit trees and vegetables from 1990 to 2017.

In contrast, the cultivation area of cash crops such as vegetables and fruits continued to increase (Figure 2). The cultivation areas of orchards and vegetables increased by 931 × 10^4^ hm^2^ and 1702.42 × 10^4^ hm^2^, respectively, which were 2.5 times and 3.2 times the initial stage, respectively. It is worth mentioning that the adjustment of agricultural structure is reversible, especially the transformation between cultivated land and garden land [32]. Therefore, the conversion of cultivated land to garden land can be regarded as reserved GPP, but the land has not yet been fully excavated. In addition, when the ploughing layer of cultivated land is converted to fishponds, the land is not destroyed, and this part of cultivated land can still be a backup source of emergency cultivated land. Studies have shown that the productivity of cultivated land after fishpond reclamation did not decrease significantly but moderately increased [32]. Therefore, it is necessary to regard cultivated land that is converted to a water surface as a kind of grain reserve when evaluating the GPP of cultivated land in China.

### 2.2. Irreversible Cultivated Land Use Change

Urban expansion occupies a large amount of cultivated land that is used for urban infrastructure construction, real estate development, and industrial and mining enterprises [10,32]. Land use transformation and engineered hardening such as the public facilities of "Seven Connections and One Levelling” are usually adopted. Therefore, cultivated land that is converted to construction land is difficult to reclaim for agricultural cultivation and is regarded as an actual grain production loss [32]. Statistics showed that approximately 300 × 10^4^ hm^2^ of high-quality cultivated land was occupied by construction land from 1996 to 2009 [10], and the loss of grain crop production caused by urban expansion was approximately 3490 × 10^4^ t from 1990 to 2010 [28]. Although the government has implemented the Dynamic Balance of Cultivated Land System [33], the momentum of construction land occupation of cultivated land has not been well controlled [34,35]. Thus, a reduction in this type of cultivated land is considered a loss of GPP because this land type is difficult to reclaim.

## 3. Materials and Methods

### 3.1. Data

The data used in this paper included land use change data, digital elevation model (DEM) data, and statistical data. First, the land-use data (1 × 1 km) were from the Resource and Environment Science Data Centre of the Chinese Academy of Sciences (http://www.resdc.cn/). Second, the agricultural data, such as the total grain yield and crop sown area, were from the China Rural Statistical Yearbook, and the social and economic data, such as gross domestic product (GDP) and the per capita disposable income of urban residents, were from the National Bureau of Statistics (http://data.stats.gov.c). Finally, the construction land area and cultivated land area data were from the service platform of land survey results sharing and the application of the Ministry of Natural Resources of China (http://tddc.mlr.gov.cn/toLogin).

### 3.2. Methods

#### 3.2.1. Estimation of the Reserved and Lost GPP Caused by Cultivated Land Use Change

The GPP of cultivated land to be excavated included recoverable cultivated land production potential and unrecoverable cultivated land production potential loss. The formula was as follows:

(1) $$CLPP=CLPP_{1}-CLPP_{2}$$

where *CLPP* is the *GPP* of cultivated land to be excavated, *CLPP_1_* is the recoverable *GPP* of cultivated land, and *CLPP_2_* is the loss of unrecoverable cultivated land grain potential.

(1) GPP of Cultivated Land That Can Be Recovered

The production potential of cultivated land that can be recovered included the GPP due to a decline in grain yield per unit, an MCI reduction, and agricultural structure adjustment. The formula was as follows:

(2) $$CLPP_{1}=\sum_{j=1}^{31}\left(Q_{j1}+Q_{j2}+Q_{j3}\right)$$

where *CLPP_1_* is the recoverable *GPP* of cultivated land; *Qj_1_* is the *GPP* due to the yield per unit area reduction in the *j^th^* province, *j* = 1, 2, …, 31; *Qj_2_* is the *GPP* due to MCI reduction in the *j^th^* province; and *Qj_3_* is the *GPP* due to agricultural structure adjustment in the *j^th^* province.

First, the reserved GPP due to a yield per unit area reduction referred to the maximum per unit area yield of grain in history achieved by using agricultural technology to increase the grain yield. Part of the increase in grain production was the reserved potential for a reduction in yield per unit area. This potential size was estimated by multiplying the yield reduction per unit area by the sown area of grain in 2016. The formula was as follows:

(3) $$Q_{j1}=\left(Y_{max}-Y_{2017}\right)\cdot S_{2017}$$

where *Q_j1_* is the GPP due to a yield per unit area reduction in the *j^th^* province, *Y_max_* is the average of the maximum yield per unit area for two years from 1990 to 2017, *Y_2017_* is the grain yield per unit area in 2017, and *S_2017_* is the grain sowing area in 2017.

Second, the reserved GPP due to MCI reduction refers to a decrease in grain production because of a decline in the MCI. To avoid large differences in the results due to different data sources, cultivated land data published by the Ministry of Natural Resources of China were used to calculate the reserved GPP. The calculation period was 2006 to 2017 for this part of the GPP because data were available starting in 2006. The reserved GPP of the decline in MCI was obtained by multiplying the amount of multiple cropping index decrease, grain sown area, and grain yield per unit area. Considering that location is the main factor affecting crop ripening, the cultivated land area in 2006 was used to calculate the change in MCI. The formula was as follows:

(4) $$Q_{j2}=\left(MCI_{max}-MCI_{2017}\right)\cdot A_{2006}\cdot Y_{2006}$$

where *Q_j2_* is the *GPP* in province *j* due to a decline in MCI; *MCI_max_* is the maximum MCI value of grain from 2006 to 2017, which is expressed by the ratio of grain sown area to cultivated land area in province *j* in that year; *MCI_2017_* is the MCI of grain in 2017; *A_2006_* is the cultivated area in 2006; and *Y_2006_* is the grain yield per unit area in 2006.

Third, the reserved GPP due to agricultural structure adjustment included the potential in economic crop land and the potential in orchards and fishponds. The potential in economic crop land was estimated by the reduction in the proportion of grain sown area. The potential in orchards and fishponds was estimated by analysing land use change in 1990 and 2015, and the area of cultivated land that was converted to other gardens and fishponds was extracted. Considering that the grain yield may decrease to a certain extent after the reclamation of garden land and fishpond water surfaces, this part of the potential value was estimated by the average grain yield of cultivated land in 1990. The formulae were as follows:

(5) $$q_{1}=\left(R_{1990}-R_{2017}\right)\cdot S_{2017}\cdot Y_{1990},$$

(6) $$q_{2}=\Delta A\cdot Y_{cl},$$

where *q_1_* indicates the potential in economic crop land; *q_2_* indicates the potential in orchards and fishponds; *R_1990_* is the proportion of sown area for grain crops in 2017; *S_2017_* is the grain sown area in 2017; *Y_1990_* is the grain yield per unit area in 1990; *ΔA* is the area of cultivated land that was transferred to orchards and ponds from 1990 to 2017; and *Y_cl_* is the average grain yield of cultivated land in 1990, and the value was 6153 kg per hm^2^ [24]. In 1990, Chongqing belonged to Sichuan Province, and, therefore, the data were replaced with the corresponding Sichuan data.

(2) Loss of GPP of Cultivated Land that is Unrecoverable

The loss of GPP of unrecoverable cultivated land referred to grain output reductions caused by the expansion and occupation of cultivated land for construction land. The formula was as follows:

(7) $$CLPP_{2}=\left(\Delta S_{1}+\Delta S_{2}\right)\cdot Y_{cl}$$

where *CLPP_2_* is the loss of GPP of non-recoverable cultivated land; *ΔS_1_* is the cultivated land area occupied by the construction land expansion from 1990 to 2017; *ΔS_2_* is the cultivated land area of the predicted time (2017~2030) when estimating the fallow scale in 2017, and *ΔS_2_* = 0; and *Y_cl_* is the average grain yield of cultivated land in 1990.

First, the cultivated land area occupied by construction land expansion from 1990 to 2017 was estimated. The area of cultivated land that was occupied by construction land expansion from 1990~2017 was divided into two periods for the estimation. In the first stage, the construction land for urban and rural construction in China increased by approximately 175.93 × 10^4^ hm^2^ according to the study of Liu et al. [36]; 81% of the newly added construction land came from cultivated land from 1990 to 1999 [36], that is, the occupied land was 142.51 × 10^4^ hm^2^. In the second stage, the average annual increase in construction land in mainland China was 55.30 × 10^4^ hm^2^ from 2000 to 2010 according to the Remote Sensing Survey and Assessment Project of the National Environmental Protection Decade (2000–2010), which was jointly conducted by the Ministry of Environmental Projection and the Chinese Academy of Sciences. Assuming that construction land maintained an average annual rate of 55.30 × 10^4^ hm^2^ and that 81% of construction land came from cultivated land, the estimation formula for the reduction in cultivated land area due to the expansion of construction land by 2017 was as follows:

(8) $$\Delta S_{1}=142.51+55.3\times 81\%\times T_{y}$$

where *ΔS_1_* indicates the area of cultivated land occupied by construction land expansion from 1990 to 2017 and *T_y_* is the time span, that is, the year to be estimated minus 2000.

Second, the cultivated land area occupied by construction land expansion from 2017 to the forecasted year was estimated. China’s urbanization rate was 58.58% in 2018. China is still in the development stage of urbanization, and, therefore, it may be difficult for the rate of construction land expansion occupying cultivated land to decrease in a short time period. To predict the grain yield loss due to the expansion of construction land occupied by cultivated land in the next 10 years, this paper used a fixed effect model to identify the key factors regarding a scale change in construction land; additionally, a driving force model was constructed to predict the cultivated land area occupied by the expansion of future construction land with reference to Chen et al. [37]. Considering that the first land use survey data published by the Ministry of Natural Resources of China were from 2009, provincial panel data of construction land expansion from 2009 to 2017 were used for the empirical analysis. Social-economic development factors, national policies, and geographical location factors were selected as driving factors with reference to the theory of urban expansion and the related literature. The statistical description of each factor is shown in Table 1, and the empirical model settings were as follows:

(9) $$y_{it}=\sum_{k=1}^{K}X_{kit}\beta_{ki}+\mu_{it}$$

where *y_it_* is the construction land area of the *i^th^* province in the *t^th^* year; *i* = 1, 2,…31; *t* represents the known year; *X_kit_* represents the observation value of the *k^th^* variable in the *i^th^* province in the *t^th^* year; *β_ki_* is a parameter to be estimated; and *μ_it_* is a random error term.

#### 3.2.2. Estimation of the Fallow Scale of Cultivated Land

On the premise of ensuring a grain self-sufficiency rate of 100%, the population carrying capacity of the existing cultivated land was compared with that of the existing population. If the population carrying capacity exceeds the existing population size, then the existing cultivated land can be properly fallowed. The fallow scale of cultivated land was calculated as follows:

(10) $$CCLP=\left(TP_{2017}+CLPP\right)/D,$$

(11) $$F_{size}=\left(\frac{CCLP}{P}-1\right)\cdot 100\%,$$

where *CCLP* represents the population carrying capacity of total cultivated land, *TP_2017_* indicates the total grain production in 2017, *CLPP* represents the GPP of China’s cultivated land to be tapped, and *D* is the per capita food consumption level. This study selected two different living standards of per capita food consumption level. First, the per capita food consumption of a comprehensive well-off society proposed by the National Food and Nutrition Advisory Committee is 437 kg per year [39]. Second, comprehensive grain consumption, such as residents’ food, industrial consumption, loss, and waste, is 424 kg per year [40]. *F_size_* indicates the scale of cultivated land available for following and *P* is the total population of China.

## 4. Results

### 4.1. Reserved GPP of Cultivated Land

#### 4.1.1. Reserved GPP Due to a Yield per Unit Area Reduction

**Figure 3:**
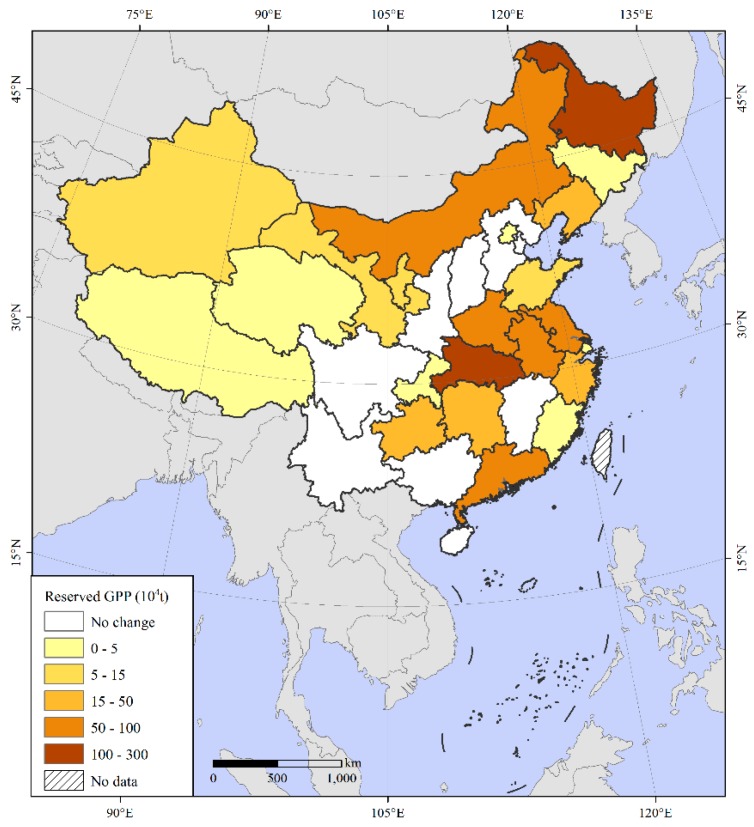
Spatial distribution of reserved grain production potential (GPP) due to a yield per unit area reduction.

Overall, the reserved GPP due to a yield per unit area reduction was 920.67 × 10^4^ t, which was mainly distributed in Northeast and East China (Figure 3). The grain yield per unit area in 22 provinces, including Beijing and Inner Mongolia, decreased from 1990 to 2017, resulting in grain reserves of different sizes. Heilongjiang had the largest reserve, 264.11 × 10^4^ t, followed by Hubei with 100.5 × 10^4^ t. In contrast, the grain yield per unit area increased in nine provinces, including Tianjin, Hebei and Sichuan. The grain increase caused by the increase in unit yield was much smaller than that caused by the decrease in unit yield; thus, reserves were formed that had not been fully exploited.

#### 4.1.2. Reserved GPP Due to Multiple Cropping Index Reduction

**Figure 4:**
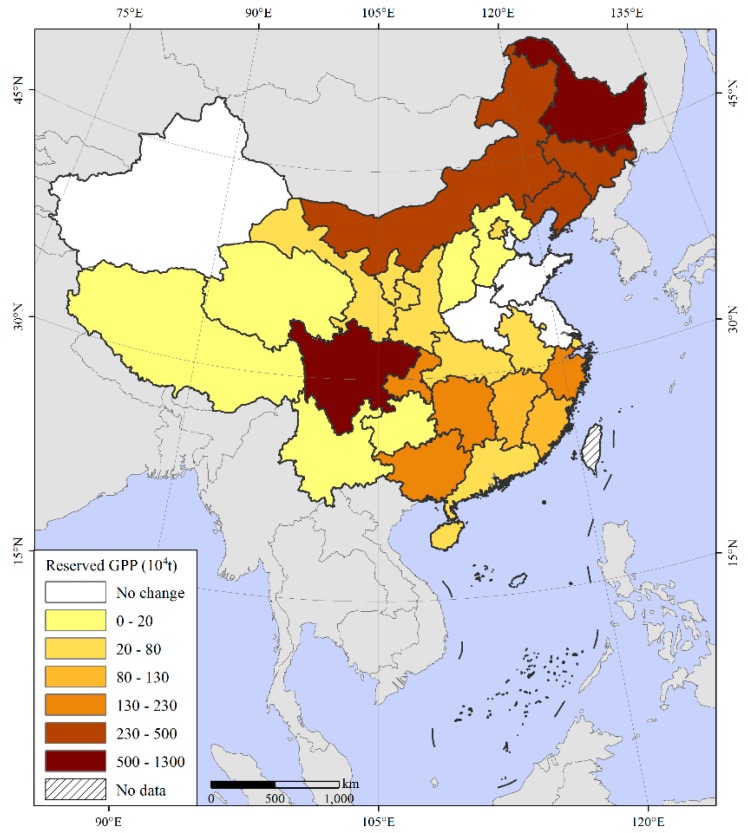
Spatial distribution of reserved GPP due to multiple cropping index reductions.

The reserved GPP due to a decline in the MCI was 4320.86 × 10^4^ t (Figure 4). Specifically, 26 provinces, including Sichuan, Inner Mongolia, Liaoning, and Jilin, formed reserves of different sizes. Sichuan Province had the largest, 1228.53 × 10^4^ t, followed by Heilongjiang Province with 941.29 × 10^4^ t. However, GPP reserves had not yet formed in some provinces, including Tianjin, Jiangsu, Shandong, Henan, and Xinjiang, as these areas did not have decreases in the MCI.

#### 4.1.3. Reserved GPP Due to Agricultural Structure Adjustment

**Figure 5:**
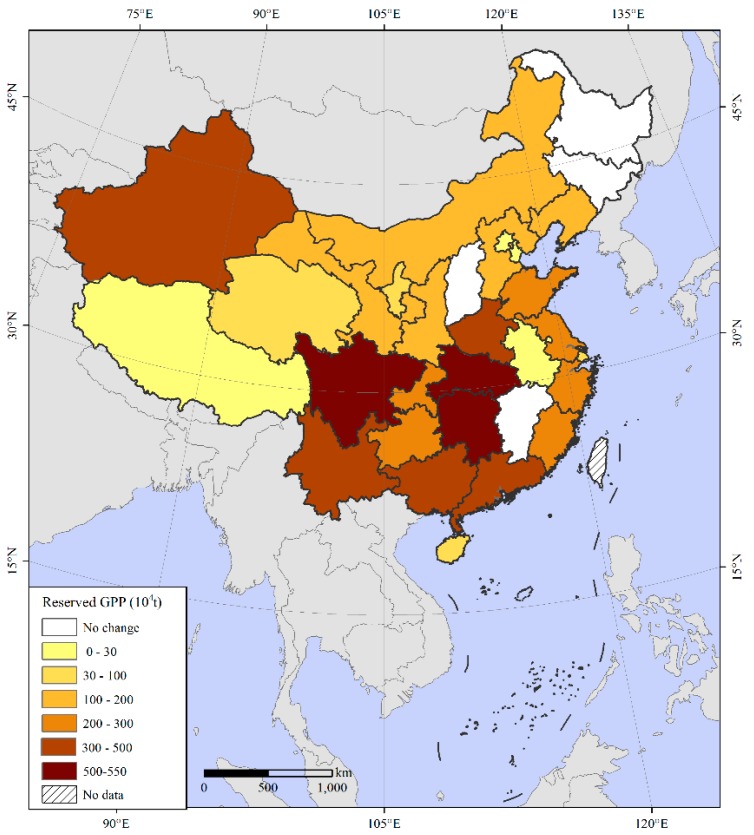
Spatial distribution of reserved GPP due to agricultural structure adjustment.

The total reserved GPP due to agricultural structure adjustment was 7759.82 × 10^4^ t, and the potential in economic crop land was 6179.91 × 10^4^ t (Figure 5). Specifically, 26 provinces, including Sichuan, Henan, Hubei, and Hunan, had different potentials, and Sichuan Province had the largest, approximately 527.97 × 10^4^ t, followed by Hubei Province with 522.65 × 10^4^ t. In contrast, Shanxi, Jilin, Heilongjiang, and Jiangxi had not yet formed reserves in economic crop land. The areas that were transferred from cultivated land to other gardens and fishponds from 1990 to 2015 were 124.56 × 10^4^ hm^2^ and 141.21 × 10^4^ hm^2^, respectively, according to the land use change matrix analysis, totalling 265.77 × 10^4^ hm^2^. The reserved GPP was 1579.91 × 10^4^ t on the basis of the average grain yield of cultivated land in 1990.

### 4.2. Lost GPP of Cultivated Land

#### 4.2.1. Lost GPP Caused by the Occupation of Construction Land from 1990 to 2017

Construction land increased by 884.8 × 10^4^ hm^2^ from 2000 to 2017, and the loss of cultivated land was approximately 716.69 × 10^4^ hm^2^ on the basis of the estimate that 81% of the construction land came from cultivated land. The total area of cultivated land occupied by the expansion of construction land from 1990 to 2017 was 859.20 × 10^4^ hm^2^, and the grain potential loss was 5286.66 × 10^4^ t during this period on the basis of the average grain yield of cultivated land in 1990.

#### 4.2.2. Predicted Lost GPP Caused by the Occupation of Construction Land under Different Economic Growth Scenarios

Before the empirical simulation, collinearity between the variables was tested. The variance inflation factor (VIF) showed that the maximum VIF of a single variable was 3.76, and the overall VIF was 4.13. These values were much less than the critical value of 10, indicating that there was no serious collinearity problem between the variables. Considering that the expansion of construction land is greatly affected by geographical location, regional dummy variables that reflected geographical location and did not change over time were included in the models. To test the robustness of the model, a random effect model was also simulated in this study because the fixed effect model automatically removed the abovementioned variables. Table 2 shows the empirical results of the key drivers of construction land expansion in the provinces and cities, and the dependent variable was construction land area. The results showed that the directions and coefficients of all drivers were consistent, indicating that the model estimation results were robust. It is worth noting that the scale of construction land expansion was affected by many factors; among them, GDP, foreign investment, policy factors, and geographical location were the key drivers.

Then, the stepwise regression method was used to identify the key drivers with a significance level within 20%. The key driving forces included GDP, per capita disposable income, foreign investment, the number of large cities, the area of cultivated land, and highway mileage. Similarly, there were no obvious collinearity problems among the factors. Next, taking the construction land area of each province as the dependent variable and key driving forces as the independent variable, the fixed effect model was used to analyse the construction land change and its driving factors. The empirical results showed that the overall F value was 77.08, the *R^2^* value between groups was 0.48, and the overall *p*-value was 0.000, indicating that the model was set up well and that the driving equation of construction land expansion was reasonable.

According to the estimated results in Table 3, the driving force equation of construction land change from 2009 to 2017 was obtained as follows:

*SCL* = −5148.21 + 0.0014*GDP* + 1.09*MH* − 3.07*NBC* + 0.024*NT* + 0.057*ACL* − 0.00006*FI* (12)

where *SCL* is the construction land area, *GDP* is gross domestic product, *MH* is highway mileage, *NBC* is the number of large cities, *NT* is the number of towns, *ACL* is the farmland area, and *FI* is total foreign investment. Subsequently, the driving force equation of construction land change was used to predict the scale of construction land expansion.

As shown in Table 3, the maximum standardization coefficient of GDP was 0.635, indicating that GDP was the most important driving force for construction land expansion in the last ten years. Therefore, this paper predicted the expansion area of construction land in the future under the scenarios of low-, medium-, and high-speed economic growth according to the change in GDP. The base period was 2017, and other factors were kept at the average level from 2009 to 2017. According to the international economic growth standard, a GDP growth rate below 3% is considered low economic growth, a growth rate between 3% and 6% is considered medium–high growth, and a growth rate between 6% and 8% is considered high-speed growth. In this paper, 3% was the low-speed economic growth rate, and 6% was the medium–high-speed economic growth rate, and the prediction results of lost GPP caused by the expansion area of construction land and its occupation of cultivated land are shown in Table 4. If other factors remain unchanged, the scale of expansion of construction land in China will be 408.10 and 1017.28 hm^2^ if the economic growth rate is estimated at 3% and 6% by 2030, and the reduction in cultivated land will be 330.56 and 824 hm^2^, respectively. Correspondingly, the actual lost GPP of cultivated land were 2033.95 and 5070.05 t.

### 4.3. Total Productive Capacity of Cultivated Land

According to the above calculation, the reserved GPP due to unit yield decline, MCI decline, and agricultural structure adjustment were 920.67 × 10^4^ t, 4,320.86 × 10^4^ t, and 7,759.82 × 10^4^ t, respectively, and the GPP loss caused by the occupation of cultivated land by construction land was 5,286.66 × 10^4^ t (Table 5). Therefore, the reserved GPP after the balance of income and expenditure was 7714.69 × 10^4^ t, and the total grain production capacity of existing cultivated land and cultivated land to be excavated in China was 64,231.19 × 10^4^ t in addition to the total grain output in 2017 (56,516.5 × 10^4^ t).

### 4.4. Fallow Scale of Cultivated Land

#### 4.4.1. Fallow Scale of Cultivated Land in 2017

According to the well-off standard of grain consumption per capita (437 kg), the largest population carrying capacity of cultivated land in China is 1.47 billion, whereas the population carrying capacity is 1.52 billion on the basis of the comprehensive per capita grain consumption standard (424 kg). In 2017, the total population of China was 1.383 billion, and the total grain productivity of cultivated land in China was slightly larger than that of the current population in China. Therefore, China can consider moderate fallowing of cultivated land in this context. Currently, the proportion of the population that China’s cultivated land can feed at its maximum potential to the total population in 2017 was between 106.28% and 109.54%. This result indicates that the cultivated land in China can feed 6.28% to 9.54% more of the total population when land use change is considered, that is, 6.28% to 9.54% of cultivated land can be fallowed. As a result, approximately 850.22 × 10^4^ hm^2^~1291.57 × 10^4^ hm^2^ of cultivated land could have been fallowed by 2017 according to the results of the Second National Land Survey, which showed that the total area of cultivated land in China was 13,538.5 × 10^4^ hm^2^.

#### 4.4.2. Fallow Scale under Different Economic Growth Scenarios

**Figure 6:**
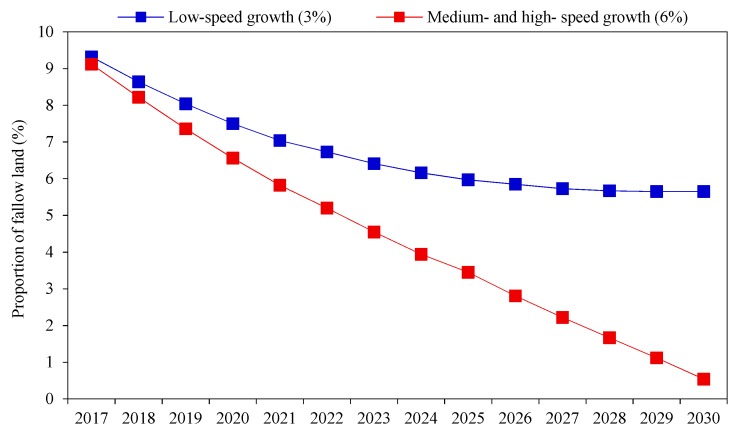
Proportion of fallow scale under different economic growth scenarios.

A population prediction model (PPM) that assumed that the fertility rate has a long-term impact on the Chinese population was used to predict the fallow scale of cultivated land [41]. Figure 6 shows the fallow scale under different economic growth scenarios in combination with the comprehensive per capita grain standard. First, in the context of low-speed economic growth, the proportion of fallow scale of cultivated land showed a downward trend from 2017 to 2030, and China could still fallow 5.65% of its cultivated land in 2030. Second, in the context of medium- and high-speed economic growth, the fallow scale proportion of cultivated land was greatly reduced. Approximately 0.54% of the cultivated land could still be fallowed under the condition that the agricultural yield increase technology did not change, but the fallow scale at this time was less than that at the initial stage.

## 5. Discussion

### 5.1. Uncertainty Analysis

There are three reasons that explain the grain production capacity of cultivated land to be excavated. First, the agricultural structure adjustment only accounted for grain yield reductions caused by the conversion of cultivated land to gardens and fishponds, and cultivated land use patterns, such as the conversion of cultivated land to grassland and forestland, was not considered. Second, the “Grain for Green Project” reduced a large amount of inferior cultivated land, resulting in an increase in grain yield and MCI in 2017 [42], and the actual reserved GPP compared with the historical amount was rather low. Third, land-use data with a 1 km resolution were used to extract the cultivated land into orchards and fishponds, and the results were limited by the accuracy of the land use data [43]. Therefore, it was difficult to identify orchards and fishponds that had small areas, which led to underestimated results. If the accuracy of land use data was improved, the underestimation problems would be eliminated.

In contrast, there were some factors that were overestimated in the empirical results regarding the potential of cultivated land to be excavated. First, the MCI value of non-grain crops was rather high, and the GPP in cash crop land was relatively large. Second, some orchards are planted by intercropping grains, which was not analysed in this paper. In addition, all the grain yield data per unit used in this study were at the provincial level with a large spatial scale, and errors would be reduced if the data were at the county level or if a smaller scale was used. Finally, in the prediction section of fallow scale, we only considered the most important driving force (GDP) changes. Indeed, the empirical results also included other drivers (e.g., GDP, NBC, and NT), which reduced the prediction accuracy to some extent. However, considering that these drivers are relatively stable, it has less impact on simulation results. 

### 5.2. Comparison of the Estimation Results

According to the Outline of the National Medium- and Long-Term Plan for Food Security (2008–2020) issued by the Chinese government in 2008, the amount of cultivated land should be no less than 12,000 × 10^4^ hm^2^ by 2020. In 2016, the Adjustment Plan for the Outline of the National Land Use (2006–2020) further stipulated that the amount of cultivated land in China should be more than 12,433 × 10^4^ hm^2^ by 2020. It was found that 850.22 × 10^4^ hm^2^ to 1291.57 × 10^4^ hm^2^ of cultivated land could be fallowed in 2017, and the remaining cultivated land that could be used for cultivation ranged from 12,246.93 × 10^4^ hm^2^ to 12,688.28 × 10^4^ hm^2^, which was basically similar to the amount of cultivated land required in the Outline of the National Land Use (2006–2020). The fallow scale of cultivated land estimated in this study is thus quite reasonable.

### 5.3. Practical Significance of Exploiting the Productive Potential of Cultivated Land

With the rapid development of urbanization, a large amount of high-quality cultivated land has been occupied. To cope with the loss of cultivated land and guarantee grain outputs, the Chinese government has implemented land improvement projects. From 2001 to 2015, the cultivated land area increased due to nationwide land improvement projects, reaching 413.5 × 10^4^ hm^2^ [24], which was converted from barren grass and other land with poor fertility, such as land with soil erosion and fragmented land [10,44]. It is noteworthy that the loss of cultivated land was mainly distributed in South China, with abundant water and heat resources, whereas the newly reclaimed cultivated land was mainly distributed in Northwest and Northeast China, with poor natural conditions and a mismatch of water and heat resources [35,45]. The potential of cultivated land in the south has not yet been fully exploited, and the newly reclaimed cultivated land is less productive [46].

Taking the change in MCI as an example, the reserve of GPP due to MCI reduction was 4320.86 × 10^4^ t in the past 30 years. By referring to the land reclamation cost conversion method from Jiang et al. [24], this part of GPP was equivalent to a loss of 702.24 × 10^4^ hm^2^ of cultivated land according to the average grain yield of cultivated land in 1990. The average quality of cultivated land occupied by construction land in China was approximately 1.18 times that of newly reclaimed cultivated land through land development and consolidation projects [11]. Therefore, the grain yield reduction caused by the decline in MCI was equivalent to the grain production capacity of 828.64 × 10^4^ hm^2^ of newly reclaimed cultivated land. The minimum cost of land reclamation is 75,000 yuan per hm^2^ [47]. If the GPP of this part of existing cultivated land is fully tapped, we will save 621.47 billion yuan in land reclamation costs.

## 6. Conclusions and Implications

With food security as the bottom line, this study systematically calculated the GPP of existing and unexcavated cultivated land in China from grain yield per unit reductions, MCI declines, agricultural structure adjustments, and construction land expansion on the basis of the perspective of land use change from 1990 to 2017. The population carrying capacity of cultivated land and the theoretical scale that could be fallowed were estimated according to different per capita grain consumption levels. The main conclusions are as follows. First, the GPP of cultivated land to be excavated in China was 7714.69 × 10^4^ t. The reserved GPP from grain yield per unit reductions, MCI declines, and agricultural structure adjustments were 920.67 × 10^4^ t, 4,320.86 × 10^4^ t, and 7,759.82 × 10^4^ t, respectively, whereas the lost GPP caused by construction land expansion was 5286.66 × 10^4^ t in the past 30 years. Second, the population carrying capacity of cultivated land in China in 2017 ranged from 1.469 to 1.515 billion on the basis of the well-off consumption standard and the comprehensive grain consumption level, that is, the grain output of cultivated land can fully meet the consumption needs of the existing population. A moderate fallowing scale could be used at this stage, and the fallow scale was between 850.22 × 10^4^ hm^2^ and 1291.57 × 10^4^ hm^2^. Finally, the lost GPP caused by the occupation of construction land differed greatly depending on economic growth rates. In the low-speed economic growth scenario, China could fallow 5.65% of cultivated land by 2030, and only 0.54% of the cultivated land could be fallowed in 2030 under the scenarios of medium- and high-speed economic growth.

At this stage, if the production potential of existing cultivated land is fully tapped, the domestic food demand can be met. This scenario would also provide an opportunity for cultivated land to be fallowed in China. With the development of urbanization, the fallow scale will decrease significantly in the future, especially in the scenarios of medium- and high-speed economic growth, and the fallow scale proportion will be only 0.54% by 2030. Moreover, the grain consumption level of residents will also continue to increase as urban–rural residents’ incomes improve, and the promotion of fallow land will also be restricted by food security. Thus, we should promote fallowing as soon as possible at this stage to ensure that the cultivated land has enough time to rest and recover to ensure food security and lay the foundation for sustainable development of fallow farming.

In addition, the government should pay attention to excavating the grain production capacity of existing cultivated land. Making full use of the GPP of cultivated land will save a substantial amount of money in land reclamation costs. Additionally, the problems of newly developed cultivated land with poor quality and fragile ecological conditions can be avoided, and the dynamic balance of cultivated land can be realized more sustainably and scientifically. Concerning grain transportation from north to south, the government should pay attention to exploiting the grain productive capacity of cultivated land in South China, which is more conducive to the spatial allocation of grain.

## Figures and Tables

**Table 1:** Statistical description of the variables.

Variable	Definition	Unit	Mean	Min	Max	S.D.
*Socio-Economic Factors*						
*GDP*	Gross domestic product	10^8^ yuan	19,039.80	441.36	80,854.91	15,702.95
*PDI*	Per capita disposable income	yuan	24,147.86	11,929.78	57,691.67	8192.75
*FAI*	Fixed asset investments in the province	10^8^ yuan	13,179.56	378.28	53,322.94	9977.71
*FI*	Foreign investment in the province	10^6^ dollars	112,423.86	534.00	879,868	166,834
*TP*	Total population of the province	10^3^	4360.86	296.00	10,999.00	2749.12
*UR*	Urbanization rate	%	53.79	22.30	89.60	13.95
*UP*	Urban population	10^3^	2332.08	66.00	7611.00	1549.15
*Policy Factors*						
*NSM*	Number of small and medium cities		5.01	0.00	10.00	3.20
*NBC*	Number of big cities		4.34	0.00	17.00	4.06
*NT*	Number of towns		564.86	73.00	1704.00	355.14
*ACL*	Area of cultivated land	10^4^ hm^2^	435.96	18.77	1586.59	3282.15
*MRS*	Mileage of railway service	10^4^ km	0.33	0.03	1.23	0.20
*MH*	Highway mileage	10^4^ km	13.83	1.17	32.41	7.42
*Geographical Location Factors*						
*CR*	In the central region		0.26	0.00	1.00	0.44
*ER*	In the eastern region		0.35	0.00	1.00	0.48
*PC*	Plain areas		0.52	0.00	1.00	0.50

Note: (1) The dummy variables in the central and eastern regions were compared with the western region. The eastern region included Beijing, Tianjin, Hebei, Liaoning, Shandong, Shanghai, Jiangsu, Zhejiang, Fujian, Guangdong, and Hainan Provinces, and the central region included Shanxi, Jilin, Heilongjiang, Anhui, Jiangxi, Henan, Hubei, and Hunan Provinces. (2) The plain and mountainous areas were determined on the basis of a digital elevation model (DEM), and mountains were mainly related to topography. According to the World Protection Monitoring Centre (UNEP-WCMC), an area can be classified as a mountain area if it meets the following conditions: (i) the elevation is between 1500 and 2500 m, and the slope is greater than 2°; (ii) the elevation is between 1000 and 1500 m, and the slope is greater than 5°, or the local height difference is over 300 m; and (iii) the elevation is between 300 and 1000 m, and the local difference is greater than 300 m [38].

**Table 2:** Results of the estimations and tests of the models.

Variable	Fixed Effect Model		Random Effect Model	
	Coefficient	T Value	Coefficient	T Value
*Socio-Economic Factors*				
*GDP*	0.0014 ***	6.15	0.001 ***	3.65
*PDI*	0.0003 *	1.86	−0.00009	−0.69
*FAI*	0.0001	0.44	0.0002	0.76
*FI*	−0.00006 ***	−4.06	−0.00004 ***	−2.70
*TP*	−0.011	−1.41	0.017 ***	5.39
*UR*	−0.004	−0.41	−0.019	−0.66
*UP*	−0.006	−0.81	−0.012 **	−2.30
*Policy Factors*				
*NSM*	−0.372	−0.49	1.535 *	1.65
*NBC*	−3.247 **	−2.14	−0.423	−0.33
*NT*	0.031 **	2.35	0.009	0.47
*ACL*	0.073 *	1.78	0.004 ***	2.63
*MRS*	−6.558	−0.57	21.425 **	1.97
*MH*	1.303 *	1.93	1.586 *	1.78
*Geographical Location Factors*				
*Eastern region*			18.223 **	2.41
*Central region*			4.287	0.45
*Plain area*			31.827 ***	3.96
*Year dummy*	yes		yes	
Constant	−179.81	−1.06	−25.429 ***	−3.17
Sigma_u	204.18		11.90	
Number of observations	279		279	

Note: *, **, and *** are significantly different from zero at the 10%, 5%, and 1% levels, respectively.

**Table 3:** Simulation results of the key drivers of construction land expansion.

Variable	Coefficient	Std. Error	T value	*p* > |t|	Standardization Coefficient
*GDP*	0.0014 ***	0.0002	6.08	0.000	0.635
*PDI*	0.0003	0.0002	1.58	0.124	0.126
*FI*	−0.00006 ***	0.00001	−4.33	0.000	0.211
*NBC*	-3.066 **	1.452	−2.11	0.043	0.260
*NT*	0.024 **	0.011	2.14	0.041	0.110
*ACL*	0.057 *	0.031	1.83	0.077	0.328
*MH*	1.091 *	0.587	1.86	0.073	0.185
*Constant*	−5148.210	139.332	−1.19	0.243	
*Sigma_u*	158.513				
*Sigma_e*	7.003				
*Rho*	0.998				
*Number of observations*	279				

Note: (1) *, **, and *** are significantly different from zero at the 10%, 5%, and 1% levels, respectively. (2) Standard error has been clustered to provincial scale, which can reduce the influence of heteroscedasticity. In addition, Taking logarithm of the variables measured in currency also reduced the influence of heteroscedasticity to some extent. On the whole, the influence of heteroscedasticity is small and can be ignored.

**Table 4:** Expansion area of construction land and loss of GPP (2017~2030).

	Low-Speed Economic Growth				Medium–High-Speed Economic Speed			
Year	GDP (10^8^ yuan)	Construction Land Expansion Area (10^4^ hm^2^)	Occupied Cultivated Land Area (10^4^ hm^2^)	Lost GPP (10^4^ t)	GDP (10^8^ yuan)	Construction Land Expansion Area (10^4^ hm^2^)	Occupied Cultivated Land Area (10^4^ hm^2^)	Lost GPP (10^4^ t)
2017	762,262.62	15.24	12.35	75.96	784,464.45	39.66	32.13	197.68
2018	785,130.50	40.40	32.72	201.33	831,532.31	91.44	74.07	455.72
2019	808,684.42	66.31	53.71	330.46	881,424.25	146.32	118.52	729.25
2020	832,944.95	92.99	75.32	463.47	934,309.71	204.49	165.64	1019.18
2021	857,933.30	120.48	97.59	600.46	990,368.29	266.16	215.59	1326.51
2022	883,671.30	148.79	120.52	741.57	1,049,790.39	331.52	268.53	1652.28
2023	910,181.44	177.95	144.14	886.90	1,112,777.81	400.81	324.65	1997.60
2024	937,486.88	207.99	168.47	1036.60	1,179,544.48	474.25	384.14	2363.64
2025	965,611.49	238.93	193.53	1190.79	1,250,317.15	552.10	447.20	2751.64
2026	994,579.83	270.79	219.34	1349.60	1,325,336.18	634.62	514.04	3162.91
2027	1,024,417.23	303.61	245.93	1513.18	1,404,856.35	722.09	584.90	3598.87
2028	1,055,149.74	337.42	273.31	1681.67	1,489,147.73	814.82	660.00	4060.98
2029	1,086,804.23	372.24	301.51	1855.21	1,578,496.59	913.10	739.61	4550.82
2030	1,119,408.36	408.10	330.56	2033.95	1,673,206.39	1017.28	824.00	5070.05

**Table 5:** Reserves and losses of GPP of cultivated land to be excavated.

Category	Grain Production Capacity (10^4^ t)	Ratio of Grain Output in 2017 (%)
*Reserved GPP*		
Per unit yield reduction	920.67	1.63
Multiple cropping index reduction	4320.86	7.65
Agricultural structure adjustment	7759.82	13.73
Transfer to economic crops	6179.91	10.93
Transfer to orchard and fishponds	1579.91	2.80
*Lost GPP*		
Occupation of construction land	−5286.66	−9.35
Total	7714.69	13.65
